# Sustainable Deforestation Evaluation Model and System Dynamics Analysis

**DOI:** 10.1155/2014/106209

**Published:** 2014-08-28

**Authors:** Huirong Feng, C. W. Lim, Liqun Chen, Xinnian Zhou, Chengjun Zhou, Yi Lin

**Affiliations:** ^1^College of Transportation and Civil Engineering, Fujian Agriculture and Forestry University, Fuzhou, Fujian 350002, China; ^2^Shanghai Institute of Applied Mathematics and Mechanics, Shanghai University, Shanghai 200072, China; ^3^Department of Architecture and Civil Engineering, City University of Hong Kong, Kowloon, Hong Kong; ^4^Department of Mechanics, Shanghai University, Shanghai 200444, China; ^5^Shanghai Key Laboratory of Mechanics in Energy Engineering, Shanghai University, Shanghai 200072, China

## Abstract

The current study used the improved fuzzy analytic hierarchy process to construct a sustainable deforestation development evaluation system and evaluation model, which has refined a diversified system to evaluate the theory of sustainable deforestation development. Leveraging the visual image of the system dynamics causal and power flow diagram, we illustrated here that sustainable forestry development is a complex system that encompasses the interaction and dynamic development of ecology, economy, and society and has reflected the time dynamic effect of sustainable forestry development from the three combined effects. We compared experimental programs to prove the direct and indirect impacts of the ecological, economic, and social effects of the corresponding deforest techniques and fully reflected the importance of developing scientific and rational ecological harvesting and transportation technologies. Experimental and theoretical results illustrated that light cableway skidding is an ecoskidding method that is beneficial for the sustainable development of resources, the environment, the economy, and society and forecasted the broad potential applications of light cableway skidding in timber production technology. Furthermore, we discussed the sustainable development countermeasures of forest ecosystems from the aspects of causality, interaction, and harmony.

## 1. Introduction

Sustainable development is an agreed-upon global forestry trend because it impacts other deforestation-related industries. Lots of factors, such as forest canopy density, forest degradation, the patterns and processes of deforestation, and logging intensity, are important for the sustainable development of forest ecosystems and the global carbon budget [1–8]. The important principles of Chinese forestry blueprinting have been well integrated with the harnessing of natural resources, environmental protection, and ecological balance. Improving the ecological environment, focusing on ecoengineering, and effectively maintaining ecology have become the leading demands of socioeconomic forestry development in this century [1–8]. The change of this leading demand has given the Chinese forestry industry the most favorable economic development position. Specifically, forestry has become the center and foundation of ecological maintenance and socioeconomic sustainable development. Forestry is no longer a single industry; rather, it is a comprehensive and dynamic system within which any development or change of its components would directly or indirectly impact the entire economy.

Therefore, in response to sustainable forestry development, a broad set of regulations, guidelines, and the technology were developed to control and safeguard forest management practice that encompasses silvicultural treatment, forest conservation strategy, cutting rate control, deforestation and forest degradation monitoring, developing cableway logging [9–12], reducing impact logging, assessment of biomass and carbon stock, and so forth [12]. Many studies have examined the patterns and processes of deforestation [6–8, 13–17], but information about the light cableway skidding technology beneficial to forest ecosystems is still limited [10, 18–25].

Noticeably, technological innovation is one of the most important components of the forestry system. Scientific deforestation plays a vital role in the forestry sustainable development and forestry competitiveness. The adoption of deforestation technology may have a direct or an indirect and beneficial or damaging impact on the overall forestry system; furthermore, it also has immediate implications for the sustainable development of forest resources as well as the economy and society. Accordingly, the engineering-based study of the adoption of rational analytic methods for the evaluation of sustainable forestry development is of great significance.

Thomas firstly proposed Analytic Hierarchy Process (AHP) which was more than one-index synthesis evaluating method [26]. AHP has been applied widely in multicriteria selection, risk evaluation, economic valuation, planning and resource allocation, and conflict resolution, et al. [27, 28]. And then, the Fuzzy Analytic Hierarchy Process (FAHP), which combined AHP with fuzzy numbers reduces, was developed. But the subjective factor could influence evaluation when using this model to a certain degree. Therefore, improvement of FAHP method to construct a sustainable deforestation development evaluation system and an evaluation model of sustainable deforestation development is necessary.

This paper presents a qualitative and quantitative analysis of the dynamic interaction of every factor in the system, with the improved Fuzzy Analytic Hierarchy Process (improved FAHP), to explore its impacts on the sustainable forestry development by combining the light cableway skidding and the traditional road-skidding in the design validation and usability testing process.

## 2. Fundamental Principles of the Evaluation Model

The forest ecosystem is characterized by systematization, comprehensiveness, diversification, connectivity, interactivity, and compatibility, which complicates the construction of the evaluation model and the index system. Therefore, after performing an insightful literature review to evaluate forestry competitiveness, we propose that an evaluation model for sustainable deforestation development and dynamic system should adhere to the following fundamental principles.

### 2.1. Systematization and Connectivity

The forest ecosystem is complicated and features systematization and comprehensiveness, which has many interrelated and interacted components. This system is also interlocked with every operation of forestry business, demonstrating its innate diversification. Noticeably, every subsystem in the forest ecosystem is not only relatively independent but also interdependent. They are directly or indirectly related to each other to develop dynamically and to compose a compatible system.

### 2.2. Scientific Integrity and Objectivity

Scientific integrity refers to the fact that research in any discipline should make its subject objective, use detectable laws, and have theoretical verifiability, strict logicality, and united scientific value. Specifically, the construction of the model should not only conform to the fundamental scientific principles but also reflect the internal components, law of development, and characteristics of the material and technical base of the forest ecosystem itself. Additionally, the construction of the evaluation model and the choosing of an index system should consider the architecture layer, organization, and archetypes. Objectivity means that the construction of the evaluation model and the chosen index should be as consistent as possible with the objective reality and the objective laws of forest ecosystem development. In addition, all of the data from the index and evaluation systems should be as objective and accurate as possible. The data collection process should be based on the statistics released by national or provincial statistics departments or profession-qualified statistics institutions to guarantee data authority and reliability.

### 2.3. Sustainable Development

Because the forest ecosystem has economic, ecological, and social benefits, forest operators cannot be driven merely by economic interests but must be guided by a sustainable development principle to reconcile these main three benefits. Additionally, forest operators should be oriented by the market mechanism and the law of value reasonably and should consider the economic and social sustainable development of the forest in the pursuit of profits. Hence, the related governmental departments should provide necessary guidelines, education, and regulatory supervision to ensure that the forest operators are engaged in sustainable development efforts.

### 2.4. Transparency

Transparency means that the construction of the evaluation model and index system for the sustainable forest development is set to be open, transparent, accurate, and specific so that some constructive suggestions can be adopted.

### 2.5. Reliability

Reliability requires that the methods adopted by the evaluation model for sustainable forest development should be feasible and practical. Otherwise, the model would lose its significance and operability. The index system should have a reliable, continuous, and authoritative data source. Since some important indexes fail to attain reliable data sources, they should be preserved until the data collection process is ready or they are just regarded as a theoretical basis and the calculation is omitted. On all accounts, the index system should represent the facts and be precise, workable, and practical.

### 2.6. Comparability

Comparability means that the construction of the evaluation model and index system for the sustainable forest development should be available for objective comparison with the alternatives. Different types of statistics are used to reflect the sustainable development of the forest during construction; so, different types of indexes should be compared. For example, the dimensionless method can give index comparability.

### 2.7. Qualitative versus Quantitative Analysis

In the sustainable forestry development system, the interaction of every component demonstrates its complexity, comprehensiveness, connectivity, and compatibility. Therefore, in the specific evaluation process, qualitative analysis should be applied to evaluate the objectives, construct a scientific and feasible index evaluation model, and use specific data to assess the state of the sustainable development. Only in this fashion can the scientific integrity, objectivity, and sustainability of the evaluation system be obtained. Therefore, quantitative analysis cannot ignore the significance of qualitative analysis, and actually, the related index of the sustainable forestry development cannot be fully quantified. Instead, necessary notes and explications should be added to specify the definition, function, and equations of every index. In the evaluation practice, the combination of qualitative and quantitative analysis makes scientific evaluation possible.

## 3. Flow Chart of the Causal Power System

**Figure 1:**
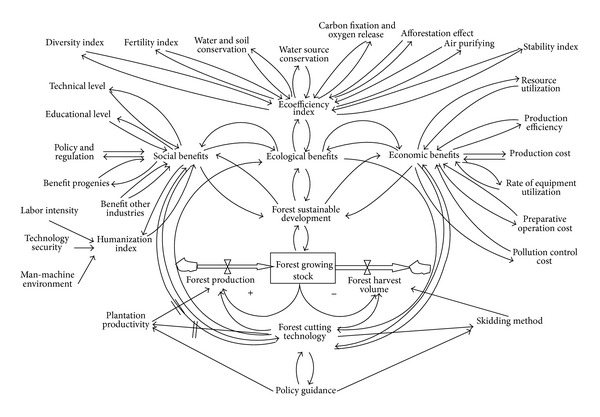
A causal power flow diagram showing the parameters and dynamics of sustainable deforestation development.

By drawing on the competitiveness of China's provincial forestry development report and literature, we compared the analysis of the impact of forest harvesting on the benefits and evaluation model of forest ecological harvesting and other related documents [12]. We elected the primary index of the three foresting benefits and contacted seven relevant experts in these fields (one in forestry, two in forest engineering, two in forest cultivation, and two in forest management) to leverage the expert knowledge and experience and determine the index system of forest harvesting to ensure sustainable forest development. The sustainable development of forestry causality and power flow graph and its index system are shown in Figure 1, while the process used to determine each index factor is analyzed and shown properly (Table 1).

In view of the system dynamics causal flow diagram's intuitive and system characteristics [10, 12, 29, 30], the forest ecological, social, and economic benefits are relatively independent and mutually influential (Figure 1). The policy guidance of forest harvesting technology influences the starting point, the contact and interaction system of sustainable forestry development influence. Figure 1 shows that forest management policy guidance and regulations guiding forest harvesting techniques, which affects forest land productivity, felling intensity, forest prodiction, and forest harvesting. According to forest stock volume, furthermore, the effect include the time lag of the forest production effect [31–34], are not directly reflected. However, the cutting technology of forests has large ecological, social, and economic benefits. For example, according to the characteristics of forest terrain, high mountains, steep slope, and rainy climate, being forced to use road-cutting skidding is bound to cause soil erosion and loss and to deplete natural resources. If the latter uses large amounts of nitrogen and phosphorus, large quantities of chemical fertilizers will enter the surface water, travel to rivers and lakes, and create an eutrophication problem that negatively affects plant and animal survival. In addition, water pollution not only deteriorates surface water quality but also has a significant impact on groundwater quality. Chemicals used in forestry production inevitably enter the groundwater supply. Therefore, the different harvesting methods affect biodiversity, fertility, soil and water conservation, carbon fixation and oxygen release, and air purification and impact the ecological, social, and economic benefits.

## 4. Improved Fuzzy Analytic Hierarchy Process (FAHP)

Analytic Hierarchy Process (AHP) is proposed firstly by Thomas L Saaty and is more than one-index synthesis evaluating method [26]. This is made possible by taking several factors into consideration simultaneously, allowing for dependence and for feedback and making numerical tradeoffs to arrive at a synthesis or a conclusion. It offers psychologists, scientists, sociologists, political scientists, and researchers the methodology they have sought for some time to quantify and derive measurements for intangibles [35, 36]. The AHP is used to derive ratio scales from both discrete and continuous paired comparisons in multilevel hierarchic structures and is concerned with providing people in the physical and engineering sciences with a quantitative method to link hard measurement to human values, which combines qualitative and quantificational methods and guarantees the system and rationality of the model, while reducing the subjective factor's effect based on personnel valuable experience and judgment [37–39]. So, it has found its widest applications in multicriteria selection, risk evaluation, economic valuation, planning and resource allocation, and conflict resolution, et al. [27, 28]. Fuzzy Analytic Hierarchy Process combined FAHP with fuzzy numbers reduces, to a certain degree, the subjective factor to evaluation influence and the application which puts the improved FAHP method to construct a sustainable deforestation development evaluation system and an evaluation model of sustainable deforestation development.

An improved FAHP uses the three-scale division belonging to the complementary scaling according to its metrics to compare the relationships among the elements. This method is accurate due to its very design. The purpose of the judgment matrix to transform into fuzzy consistent matrix satisfies the consistency condition, so there is no need to verify its consistency. The square root method and normalized method target weight *W* = (*ω*_1_,*ω*_2_,…,*ω*_*n*_)^*T*^ as the eigenvalue method of iterative initial value *V*_0_ = *V*_0_(*v*_01_,*v*_02_,…,*v*_0*n*_)^*T*^ can greatly reduce the number of iterations, improve the convergence speed, and meet the precision requirement [36, 38, 40–44]. The specific steps are as follows.

### 4.1. Establish the Priority of Judgment Matrix

Table 2 shows the three-scale division and the seven experts' opinions and judges the factors from the target layer (first layer) and the criterion layer (layer second) *A* − *B*_*i*_; then, it compares the relative importance of the factors to produce a priority judgment matrix (Table 2). To simplify the calculation, the priority factor judgment matrix corresponding to the seven experts was made on a scale ≥3 according to expert evaluation. For example, the seven experts' evaluation results were 0, 0, 0.5, 1, 0.5, 0, and 1, and since three experts assigned 0, it is used for *a*_*ij*_ and then, corresponding to the evaluation between *B*_*i*_ (Table 3), the fuzzy judgment matrix (dual priority judgment matrix) *F* is used:

(1) $$\begin{array}{c} F=\left[\begin{array}{ccc} 0.5 & 1 & 1\\ 0 & 0.5 & 1\\ 0 & 0 & 0.5 \end{array}\right]. \end{array}$$

### 4.2. Construct Fuzzy Consistent Judgment Matrix

Calculating the *r*_*i*_ = ∑_*j*=1_^*n*^ *f*_*ij*_ based on (1), we can get *r*_1_ = *f*_11_ + *f*_12_ + *f*_13_ = 0.5 + 1 + 1 = 2.5, *r*_2_ = *f*_21_ + *f*_22_ + *f*_23_ = 0 + 0.5 + 1 = 1.5, and *r*_3_ = *f*_31_ + *f*_32_ + *f*_33_ = 0 + 0 + 0.5 = 0.5. Then, we use the conversion formula *r*_*ij*_ = (*r*_*i*_ − *r*_*j*_)/2*n* + 0.5 to transform the fuzzy judgment matrix *F* = (*f*_*ij*_)_*n*×*n*_ to the fuzzy consistent judgment matrix *R* = (*r*_*ij*_)_*n*×*n*_. Thus we can get *R*

(2) $$\begin{array}{c} R=\left[\begin{array}{ccc} r_{11} & r_{12} & r_{13}\\ r_{21} & r_{22} & r_{23}\\ r_{31} & r_{32} & r_{33} \end{array}\right]=\left[\begin{array}{ccc} 0.5 & 0.667 & 0.833\\ 0.333 & 0.5 & 0.667\\ 0.167 & 0.333 & 0.5 \end{array}\right], \end{array}$$

where

(3) $$\begin{array}{c} n=3,\\ r_{11}=\frac{r_{1}-r_{1}}{2\;\times \;3}+0.5=\frac{2.5-2.5}{2\;\times \;3}+0.5=0.5,\\ r_{12}=\frac{r_{1}-r_{2}}{2\;\times \;3}+0.5=\frac{2.5-1.5}{2\;\times \;3}+0.5=0.667,\\ r_{13}=\frac{r_{1}-r_{3}}{2\;\times \;3}+0.5=\frac{2.5-0.5}{2\;\times \;3}+0.5=0.833,\\ r_{21}=\frac{r_{2}-r_{1}}{2\;\times \;3}+0.5=\frac{1.5-2.5}{2\;\times \;3}+0.5=0.333,\\ r_{22}=\frac{r_{2}-r_{2}}{2\;\times \;3}+0.5=\frac{1.5-1.5}{2\;\times \;3}+0.5=0.5,\\ r_{23}=\frac{r_{2}-r_{3}}{2\;\times \;3}+0.5=\frac{1.5-0.5}{2\;\times \;3}+0.5=0.667,\\ r_{31}=\frac{r_{3}-r_{1}}{2\;\times \;3}+0.5=\frac{0.5-2.5}{2\;\times \;3}+0.5=0.167,\\ r_{32}=\frac{r_{3}-r_{2}}{2\;\times \;3}+0.5=\frac{0.5-1.5}{2\;\times \;3}+0.5=0.333,\\ r_{33}=\frac{r_{3}-r_{3}}{2\;\times \;3}+0.5=\frac{0.5-0.5}{2\;\times \;3}+0.5=0.5. \end{array}$$

### 4.3. Calculating Priority Vector

Use of the normalizing or square root method can obtain ranking vector as follows:

(4) W(0)=(ω1,ω2,…,ωn)T=(∑j=1nr1j∑i=1n∑j=1nrij,∑j=1nr2j∑i=1n∑j=1nrij,…,∑j=1nrnj∑i=1n∑j=1nrij)T=(0.4444,0.3333,0.2222)T,

where

(5) n=3,∑j=1nr1j=∑j=13r1j=r11+r12+r13=0.5+0.667+0.833=2,∑j=1nr2j=∑j=13r1j=r21+r22+r23=0.333+0.5+0.667=1.5,∑j=1nr3j=∑j=13r3j=r31+r32+r33=0.167+0.333+0.5=1.0,∑i=1n ∑j=1nrij=r11+r12+r13+r21+r22 +r23+r31+r32+r33=0.5+0.667+0.833+0.333+0.5 +0.667+0.167+0.333+0.5=4.5,ω1=24.5=0.4444,ω2=1.54.5=0.3333,ω3=1.04.5=0.2222,W(0)=(ω1,ω2,…,ωn)T=(∏j=1nr1jn∑i=1n∏j=1nrijn,∏j=1nr2jn∑i=1n∏j=1nrijn,…,∏j=1nrnjn∑i=1n∏j=1nrijn)T=(0.4543,0.3347,0.2110)T,

where

(6) n=3,∏j=1nr1jn=r11r12r133=0.5×0.667×0.8333=0.6525,∏j=1nr2jn=r21r22r233=0.333×0.5×0.6673=0.4807,∏j=1nr3jn=r31r32r333=0.167×0.333×0.53=0.3030,∑i=1n∏j=1nrijn=∏j=1nr1jn+∏j=1nr2jn+∏j=1nr3jn=0.6525+0.4807+0.3030=1.4362,ω1=0.65251.4362=0.4543,ω2=0.48071.4362=0.3347,ω3=0.30301.4362=0.2110.

### 4.4. Calculating the Reciprocal Matrix

The conversion formula *e*_*ij*_ = *r*_*ij*_/*r*_*ji*_ of the complementary judgment matrix *R* = (*r*_*ij*_)_*n*×*n*_ is used in the reciprocal matrix *E* = (*e*_*ij*_)_*n*×*n*_:

(7) $$\begin{array}{c} E=\left[\begin{array}{ccc} 1 & 2.0030 & 4.9880\\ 0.4993 & 1 & 2.0030\\ 0.2005 & 0.4993 & 1 \end{array}\right], \end{array}$$

where *n* = 3,

(8) $$\begin{array}{c} e_{11}=e_{22}=e_{33}=\frac{r_{11}}{r_{11}}=\frac{r_{22}}{r_{22}}=\frac{r_{33}}{r_{33}}=1,\\ e_{12}=\frac{r_{12}}{r_{21}}=\frac{0.667}{0.333}=2.0030,\\ e_{13}=\frac{r_{13}}{r_{31}}=\frac{0.833}{0.167}=4.9880,\\ e_{21}=\frac{r_{21}}{r_{12}}=\frac{0.333}{0.667}=0.4993,\\ e_{23}=\frac{r_{23}}{r_{32}}=\frac{0.667}{0.333}=2.0030,\\ e_{31}=\frac{r_{31}}{r_{13}}=\frac{0.167}{0.833}=0.2005,\\ e_{32}=\frac{r_{32}}{r_{23}}=\frac{0.333}{0.667}=0.4993. \end{array}$$

### 4.5. The Ranking Vector Using the Iterative Method for High Accuracy

Use the ranking vector *W* ^(0)^ as the eigenvalue method iterative initial value *V*_0_ which is to find the high precision of vector *W* ^(*k*)[4]^. *V*_0_ = *V*_0_(*v*_01_,*v*_02_,…*v*_0*n*_)^*T*^ = (0.444,0.333,0.222)^*T*^ is the iterative initial value, iteration by iteration formula *V* ^(*k*+1)^ = *EY* ^(*k*)^, *Y* ^(*k*)^ = *V* ^(*k*)^/||*V* ^(*k*)^||_*∞*_, *k* = 1,2,…. After four iterations, it meets the accuracy requirement, and precision is within 0.0001, so we calculate the feature vector *V*_4_ = (3.0061,1.3948,0.6476)^*T*^ and the infinite norm ||*V*_4_||_*∞*_ = 3.0061 = *λ*_max⁡_ and normalize *V*_4_. Consider *V*_*k*+1_ = (*v*_*k*+1,1_/∑_*i*=1_^*n*^ *v*_*k*+1,*i*_, *v*_*k*+1,2_/∑_*i*=1_^*n*^ *v*_*k*+1,*i*_,…, *v*_*k*+1,*n*_/∑_*i*=1_^*n*^ *v*_*k*+1,*i*_)^*T*^. The vector *W* ^(*k*)^ = *V*_*i*+1_ is the ranking vector *W* ^(4)^ = (0.5954,0.2763,0.1283)^*T*^. The iteration then ends. Otherwise, *V*_*k*_ = *V*_*k*+1_/||*V*_*k*+1_||_*∞*_ = (*v*_*k*+1,1_/||*V*_*k*+1_||_*∞*_, *v*_*k*+1,2_/||*V*_*k*+1_||_*∞*_,…, *v*_*k*+1,*n*_/||*V*_*k*+1_||_*∞*_)^*T*^ for a new initial value of iteration again.

### 4.6. Calculating Layer Fuzzy Weight

According to Sections 4.1 to 4.5 above, calculating from the top to the bottom, the *B*_1_ − *C*_*i*_, *B*_2_ − *C*_*i*_, *B*_3_ − *C*_*i*_, and *C*11 − *D*_*i*_ by experts serve as factors according comparative evaluation (Table 1).  Table 4 (Table 4) lists the evaluation of *C*11 from *D*1–*D*5, and the weight of all factors of empathy can be calculated (Table 1).

## 5. Sustainable Deforestation Evaluation Model and System

### 5.1. Forest Harvesting Index

Determining the sustainable development evaluation index weight is both important and complicated. Based on the flow diagram and structure shown in Figure 1 of the different targets using a combination of quantitative and qualitative methods, if some are difficult to quantify and do not significantly influence the indicators, we can delete them. We must make full use of quantitative research results which are reported to be valid. The index calculation method mentioned in Section 4 will be used here as well as weights of all indicators listed in Table 1.

### 5.2. Evaluating the Index Calculation Formula

From the literature [45–48], combined with case (in Section 6), the new index calculation formula can be structured and the quantitative method can be used to calculate the original index value. The main index formula of the solution layer part was described elsewhere [12, 29, 30], explained as follows.

In Table 1, the integrity of the forest landscape is described by the equation *D*_1_ = *VOR*, where *V* is the vitality of the system and *V* is the main standard of metabolism and the primary productivity; *O* is a system organization index, the relative degree of which is represented by 0~1 and includes the organizational diversity and stability; *R* is the restoring force index, the relative degree of which is represented by 0~1. The diversity index is described as *D*_2_ = *N*(*N* − 1)/∑_*i*=1_^*m*^ *n*_*i*_(*n*_*i*_ − 1), where *D*_2_ is the Simpson diversity index; *N* is the total number of individuals; *n*_*i*_ is the individual number of the *i* species; *m* is the total number of species. Plantation productivity is described by the equation *D*_3_ = *P*_*s*_ + *P*_*b*_ + *P*_*l*_ + *P*_*r*_ + *P*_*n*_ + *P*_*u*_ + *P*_*h*_, where *P*_*s*_, *P*_*b*_, *P*_*l*_, and *P*_*r*_ represent the annual net growth rates of the tree trunks, branches, leaves, and roots, respectively; *P*_*n*_ is the annual litter amount; *P*_*u*_ is the annual net growth of the understory plants; *P*_*h*_ is the annual intake of the animals. The fertility index is defined by the forest restoration force *D*_4_, *D*_4_ = *M*_*s*_/*F*_*t*_ where *M*_*s*_ is the time that it takes to return from the stress state to the steady state; *F*_*t*_ is the maximum cutting intensity that the forest can bear. The stability index is shown by *D*_5_ = −∑_*i*=1_^*s*^ *r*_*i*_ln⁡*r*_*i*_, where *r*_*i*_ = *n*_*i*_/*N* represents the relative abundance of the *i* species.

Water source conservation is described by the equations *D*_6_ = *D*_6*a*_ + *D*_6*b*_, *D*_6*a*_ = ∑_*i*=1_^*n*^ *m*_*i*_(*H*_*i*_ − *H*_0_)*bβ*, and *D*_6*b*_ = *T*(*P*_1_ *a* + *P*_2_ *η*), where *D*_6*a*_ is the economic value of the forest benefit of flood control; *m*_*i*_ is the first *i* types of forest area; *H*_*i*_ is the first *I* forest type or flood storage capacity; *H*_0_ is the flood storage capacity of nonforest land; *b* is the construction fee of reservoir or dam to retain 1 m³ of the flood; *β* is the ratio of benefit/input, *D*_6*b*_ is the utilization efficiency of water resources for the forest, and *T* is the incremental amount of water resources utilization that the forest provides; *P*_1_ and *P*_2_ represent the price of irrigation and industrial water supply, respectively; *a* and *η* represent the utilization coefficient of the irrigation and industrial water supply, respectively.

Soil and water conservation is described by the equation *D*_7_ = *KP*_*r*_/2, where *K* is the amount of soil that forest land loses less than the no forest land; *P*_*r*_ is the average cost of digging 1 t sediment. Air purifying is calculated as *D*_8_ = *MLdP*_*y*_(1 + *Z*)*K*_*l*_ to measure and evaluate the forest oxygen benefit; here, *d* is the mass of dry wood; *P*_*y*_ is the price of industrial oxygen per m^3^; *Z* is the percentage of root and shoot annual growth in timber annual growth calculated by dry mass; *K*_*l*_ is the amount of oxygen emission that produces 1 t of dry matter; *M* is the stocking volume of existing forest; *L* is the net growth rate of the forests. The benefits of soil improvement are calculated by the equation *D*_9_ = *GS*_1_∑_*i*=1_^3^ *P*_1*i*_ *P*_2*i*_ *P*_3*i*_, where *G* is the amount of a type of forest's litter per unit area in a year t/(hm^2^a) and *S*_1_ is the area of a forest. The value of benefits for future generations can be defined by *D*_30_ = ∑_*i*=1_^*m*^ *N*(*n*_*i*_/*n*)*x*_*i*_, where *m* represents that the willingness to pay is divided into *m* grades; the voluntary payment of the first rank is 0; *x*_*i*_ is the voluntary payment of *i* level average (yuan/person); *N* is the total number of the investigations; *n*_*i*_ is the number of people of the *i* level.

Some of the formulae above are difficult to determine, while others require more time. Therefore, it is important to collect the expert assessment and provide data according to the formulas. It basically does not affect the qualitative analysis results; so, further accurate quantitative analyses must be performed. The unit cost of preparing the work (*D*_14_) and other experimental data are obtained from the experimental data of two kinds of cutting modes. The qualitative indexes of science and technology level and the reduction of labor intensity are divided into five grades that are directly defined by the experts' assessments, taking the standard average value and setting the standard as follows: excellent (0.80–1.0), good (0.60–0.79), middle (0.40–0.59), poor (0.20–0.39), and very poor (0.00–0.19). Value added per year (*D*_20_) and other indicators of forestry production are subjected to the forestry bureau according to the local county (city) level statistical and standardized data (Table 1).

### 5.3. Dimensionless Evaluation Index

The main purpose of the evaluation of sustainable development of forestry is choosing different indexes to reflect a certain factor's effect (e.g., cutting way) on the sustainable development of forestry. Due to the system complexity, the connotation and forms of each index in the evaluation system differ. The dimensions between indexes are difficult to reconcile. The difference is so huge that the values cannot be directly integrated together, resulting in a serious evaluation effect. Therefore, to improve the comparability of the various indicators, it is necessary to make the original data dimensionless through standardization. This study uses the threshold and fractional scaling methods of standardization to make the four indicators of the original value dimensionless. The threshold method should be divided into a positive index (i.e., the bigger the better) and a contrarian index (i.e., the smaller the better) with different transformation forms. The calculation formula is as follows:

(9) $$\begin{array}{c} y_{i}=\frac{\left[x_{i}-\min\left(x_{i}\right)\right]}{\left[\max\left(x_{i}\right)-\min\left(x_{i}\right)\right]}. \end{array}$$

For the contrarian indicator, we should first make the original data negative and then handle it without dimension using the threshold formula. The forest area data can be treated with the calibration method of standardization to express the data that have already been expressed as a percentage of data to decimal, which can serve as a data processing standard.

### 5.4. Evaluation Model of Sustainable Forestry Development

The power flow diagram (Figure 1) has the advantage of drawing the system dynamics model from the aspect of causal analysis and power utilization. However, since the system has too many factors, the FAHP is used to establish a more simple objective evaluation model. Therefore, according to the evaluation index system structure diagram of sustainable development of forestry (Figure 1), we can establish a target layer consisting of 3 two-level indexes and 9 three-level indexes to obtain quantitative data of 39 four-level indexes (Table 1).

Different evaluation indicators of sustainable development can be combined together using a certain algorithm to enable an overall evaluation. This is called target evaluation. From the analysis of the application of the comprehensive evaluation index at home and abroad, the comprehensive evaluation from the linear weighted and function formula is approximate to the complex nonlinear form but is simple and easy to understand. Therefore, the comprehensive evaluation of sustainable development

(10) $$\begin{array}{c} P=\overset{n}{\underset{i=1}{\sum }}x_{i}\lambda_{i}+c, \end{array}$$

where *P* is the comprehensive evaluation of sustainable development of forestry value; *x*_*i*_ is the standard values of indicators; *λ*_*i*_ is the comprehensive weight of the index; *n* is the index number; *c* is the correction value (the effect of other interference factors) in the actual evaluation, assuming the positive and negative effects of interference factors that offset each other, at which the *c* value is 0.

From Table 1, the following can be calculated:

(11) $$\begin{array}{c} P_{CS}=\sum x_{i}\lambda_{i}=0.6727,\\ P_{RS}=\sum x_{i}\lambda_{i}=0.4473. \end{array}$$

*P*_*CS*_ is the integrated assessment value of cableway skidding and *P*_*RS*_ is the integrated assessment value of road-skidding.

Therefore, cableway skidding has an obvious advantage over road-cutting skidding:

(12) $$\begin{array}{c} P_{CS}-P_{RS}=0.2254. \end{array}$$

## 6. Case Analysis: Comparative Analysis Light Cableway Skidding and Road-Cutting Skidding on the Sustainable Development of Forestry

### 6.1. General Situation of Test Area

Shaowu city is located in the northwest area of Fujian Province east of the Wuyi Mountains. Its latitude is 26°55′–27°35′ and its longitude is 117°2′–117°52′ in the upper reaches of the tributaries of Minjiang. It is located in the subtropical humid monsoon climate with rich resources and has the rain heat over the same period, and its three-dimensional mountain climate is very obvious. It has rain, rainstorm, flood, drought, severe thunderstorm, gale, occasional “May cold,” and hail in the summer. According to the characteristics of the forest resources and terrain conditions, through field investigation and scheme selection, two forestry farms, Huang Shan (downhill stone) areas of Shaowu city, were confirmed as the research object. XiaPoshi District 122, class 1-3 (area code 122-1-3) were chosen as the light cableway skidding test area with an area of 10.5 Hm^2^, a slope of 29°, and a total output of 972 m^3^. The cable on the fulcrum elevation was 514.96 m, lower fulcrum elevation was 425.98 m, cableway horizontal span is 380.33 m, height is 88.98 m, dip angle is 13.18°, and elevation was 402.52 M.

A winch machine with a walking tractor, which belongs to the agriculture and forestry dual-purpose machine category, has a simple structure that consists of a walking tractor, work roll, and frame, functioning as the prime mover. It is light, has low energy consumption (11.03 kW), and can be used to inverse slope set logging. Comparing the data listed in the experimentation area (Table 1), we can quantitatively explain the influence of two kinds of cutting methods on sustainable forestry development.

### 6.2. Comparison of Light Cableway Skidding and Road-Skidding from Three Benefits

It fully embodies the advantages of Ecological Logging cableway through the light cableway skidding. It protects the ecological environment, no need to open road. It does not destroy the mountain and has benefit for soil and water conservation. The use of light cableway skidding can improve skidding efficiency. It has large transportation volume and skidding speed. Its unit cost is low, which costs 70.64%~74.66% of winding open circuit skidding (Table 5). However, the early input of the light cableway skidding is high (approximately twice that of road-skidding). It is beneficial to ensure sustainable production of state-owned enterprises and the formation of professional teams. As long as the country takes agricultural promotion policy seriously and countries, departments, and farmers all participate in the process, light cable logging will become more accessible. The analysis of experimental data is detailed (see Tables 1 and 5). It compares two kinds of skidding ways of labor and the cost of the operation (Table 5). Although the test consists of an individual case, it is representative of other cases and provides a quantitative and qualitative analysis method.

## 7. Conclusions and Management Implications

### 7.1. The Advantages and the Potential Applications of Light Cableway Skidding

In this study, based on FAHP and the case analysis, we verify that cableway skidding has an obvious advantage over road-cutting skidding from the integrated assessment value, fitting to the general concept of sustainable forestry development (Table 1 and (12)). Cableway skidding creates only slight environmental damage. It is a connecting link between the two aspects of economy and ecology. The ropeway equipment has a clear production process and a clear division of the production team. It has economic and practical features, but the production process is complicated and has high technical requirements. Shaowu city of Fujian Province has incorporated mature technology. Road-skidding is a widely used traditional skidding system with a simple production process and low technical requirement. However, the destruction of the mountain is serious and is not conducive to ecological protection or soil and water conservation. The labor intensity and worker costs are high. Therefore, it is necessary to study suitable forest skidding methods in China from the perspective of its ecological, economic, and social benefits. We must make every effort to minimize ecological damage and logging costs. It is important that we improve work efficiency and reduce labor intensity to achieve the comprehensive benefits of this work to ecology, economy, and society.

Through the optimizing of light cableway logging equipment technology, the transportation efficiency can be further improved. The logging speed will be increased and the transportation costs will be reduced. Light skidding operations need to be safe and efficient. According to the terrain features, south steep slope of open problems, and rainy climate, ropeway has an obvious value that should be promoted. The evaluation of forest harvesting using the sustainable forestry development model fully demonstrated the advantage of light cable logging. Using a system dynamics causal flow chart can reflect the dynamic interaction of sustainable forestry development and comprehensive benefits. An analysis of the comprehensive qualitative and quantitative benefits was also performed. The results here showed that the traditional development model emphasizes the economy, but this should not be the only focus. Accordingly, an overly heavy focus on ecological construction is suitable for modern forest management methods. Sensitivity analyses on the cutting area, selective deforestation, unit volume, timber volume, terrain, skidding distance, the maintenance of biodiversity, site factors, and environmental factors are still needed in the future to verify the generalization of these results [49–51].

### 7.2. Countermeasures of Sustainable Forestry Development

Different perspectives must be considered in the implementation of sustainable forestry development. Importantly, the government functions should be transformed and a policy system of the sustainable development of forest resources should be established and adequately supported. New patterns of the sustainable development of forest resources can be designed according to available forest resources. Reasonable deforestation rates [29] and forest logging technologies and methods [52], such as cableway selective cutting technology [26, 53], must be determined to protect the forest system balance, maintain the normal biological cycle, and ensure mineral cycling and production cycles.

The sustainable development system can be inspected from the business model, management technology, policy, and benefits perspectives [12, 15, 18, 21, 40, 44, 54, 55]. Ultimately, the resources must be used efficiently to ensure environmental protection. Science and technology efforts should be developed and the business investment must be reasonable. Fertilizers must be carefully applied. It is important to pay attention to sustainable development efforts to enable sound scientific management decisions and further improvement of social service.

We must comprehensively strengthen our foundation of theory research and education in the area of sustainable forestry development. By doing so, we can improve the strategic consciousness and awareness while promoting the comprehensive and coordinated development of ecological, social, environmental, and economic factors [10, 56–58]. Additionally, the modernization of forestry technology and the continued scientific and technological progress must be vigorously promoted and the business mode of ecological forestry must be developed. Finally, ecological forest construction must be carefully planned.

The sustainable consumption of green forest products and high-value timber productions requires further promotion [12, 59–61]. We should also emphasize the construction and management of law of forest sustainable development in the establishment of sustainable development policies and the implementation of ecologically friendly practices.

## Figures and Tables

**Table 1:** Index weight distribution and results evaluation.

Two-level index (3)	Second weight	Three-level index (9)	Three- level weight	Four-level index	Four- level weight	Road-cutting skidding		Cableway skidding	
						Standard values	Integrated assessment value	Standard values	Integrated assessment value
*B*_1_ Ecological benefits (13)	0.5954	*C*_11_ Natural ecology (5)	0.2763	Integrity of forestry landscape (*D*_1_) Diversity index (*D*_2_) Plantation productivity (*D*_3_) Fertility index (*D*_4_) Stability index (*D*_5_)	0.0647 0.1665 0.3310 0.3310 0.1068	0.6600 0.7300 0.6200 0.6100 0.5300	0.0070 0.0200 0.0338 0.0332 0.0093	0.7800 0.7600 0.7900 0.6500 0.8200	0.0083 0.0208 0.0430 0.0354 0.0144
		*C*_12_ Ecological protection (4)	0.5954	Water source conservation (*D*_6_) Soil and water conservation (*D*_7_) Air purifying (*D*_8_) Soil improving (*D*_9_)	0.2046 0.3403 0.2505 0.2046	0.4700 0.3500 0.6700 0.3600	0.0341 0.0422 0.0595 0.0261	0.5800 0.6500 0.7100 0.7600	0.0421 0.0784 0.0630 0.0551
		*C*_13_ Ecological environment (4)	0.1283	Biomass of forest (*D*_10_) Defending the remaining woods (*D*_11_) Afforestation effect (*D*_12_) Vegetation coverage (*D*_13_)	0.3309 0.1794 0.2201 0.2696	0.5200 0.6800 0.4500 0.8900	0.0131 0.0093 0.0076 0.0183	0.5700 0.9600 0.9500 0.9800	0.0144 0.0132 0.0160 0.0202

*B*_2_ Economic benefits (9)	0.2763	*C*_21_ Cost of production (3)	0.3011	Unit cost for ready (100 million yuan, reverse index) (*D*_14_) Saving rate of unit cost (*D*_15_) Pollution controlled cost (100 million yuan, reverse index) (*D*_16_)	0.3106 0.4361 0.2533	0 0 0	0 0 0	1 0.29 1	0.0258 0.0105 0.0211
		*C*_22_ Production efficiency (3)	0.4536	Utilization rate of forest resources (*D*_17_) Utilization rate of equipment (*D*_18_) Per capita work efficiency (m^3^/per man) (*D*_19_)	0.4361 0.3106 0.2533	0.5600 0.7500 0.0740	0.0306 0.0292 0.0023	0.7800 0.8900 0.1120	0.0426 0.0346 0.0036
		*C*_23_ Input of production (3↑)	0.2453	Increment of value of annual forest production (10,000 yuan) (*D*_20_) The annual proportion of fixed investment (%) (*D*_21_) The annual proportion of construction investment (%) (*D*_22_)	0.2224 0.3357 0.4419	0.3000 0.1000 0.4000	0.0045 0.0023 0.0120	0.3500 0.1500 0.4000	0.0052 0.0034 0.0120

*B*_3_ Social benefits (17↑)	0.1283	*C*_31_ Forest resources (4↑)	0.2392	Forestry area (10,000 hm^2^) (*D*_23_) Unit area amount of growing stock (m^3^/hm^2^) (*D*_24_) Forestry land area (10,000 hm^2^) (*D*_25_) Structural integrity of forestry (*D*_26_)	0.1793 0.2201 0.2696 0.3310	0.6300 0.3900 0.5600 0.7900	0.0035 0.0026 0.0046 0.0080	0.6900 0.4400 0.6600 0.8700	0.0038 0.0030 0.0055 0.0088
		*C*_32_ Technological education (9↑)	0.4422	Improvement of scientific level (*D*_27_) Improvement of educational level (*D*_28_) Policies and regulations (*D*_29_) Benefit future generations (*D*_30_) Benefit other industries (*D*_31_) Numbers of professionals (*D*_32_) Ratio of trained staff to entire staff (*D*_33_) Investment of science and technology project and key laboratory (*D*_34_) Number of science and technology projects (*D*_35_)	0.1376 0.1201 0.1049 0.1614 0.1260 0.1026 0.0781 0.0804 0.0889	0.1600 0.1500 0.6700 0.2500 0.2700 0.0800 0.7800 0 0	0.0012 0.0010 0.0040 0.0023 0.0019 0.0005 0.0035 0 0	0.8600 0.8900 0.7800 0.5300 0.7000 0.1300 1.0000 0.1000 0.1000	0.0067 0.0061 0.0046 0.0049 0.0050 0.0008 0.0044 0.0005 0.0005
		*C*_33_ Humanization index (4↑)	0.3186	Reducing labor intensity (*D*_36_) Advancement of operation and technology (*D*_37_) Operation technical safety (*D*_38_) Harmony of man-machine environment (*D*_39_)	0.1931 0.3205 0.2248 0.2616	0.3500 0.3700 0.6800 0.5600	0.0028 0.0048 0.0062 0.0060	0.8900 0.9000 0.7600 0.8600	0.0070 0.0118 0.0070 0.0092

**Table 2:** Overview of the three-scale division.

Division	Definition	Illustration
0	*a*_*i*_ at a disadvantage to *a*_*j*_	*a*_*i*_ at a disadvantage to *a*_*j*_, then *f*_*ij*_ = 0
0.5	Equal importance	*a*_*i*_ and *a*_*j*_ have the same importance, then *f*_*ij*_ = 0.5
1	*a*_*i*_ prevails over *a*_*j*_	*a*_*i*_ prevails over *a*_*j*_, then *f*_*ij*_ = 1

**Table 3:** The evaluation table between *B*_*i*_.

Goal (*A*)	Ecological benefits (*B*_1_)	Economic benefits (*B*_2_)	Social benefits (*B*_3_)
Ecological benefits (*B*_1_)	0.5	1	1
Economic benefits (*B*_2_)	0	0.5	1
Social benefits (*B*_3_)	0	0	0.5

**Table 4:** Evaluation of *C*_*i*_.

Goal (*C*_11_)	*D*_1_	*D*_2_	*D*_3_	*D*_4_	*D*_5_
*D*_1_	0.5	0	0	0	0
*D*_2_	1	0.5	0	0	1
*D*_3_	1	1	0.5	0.5	1
*D*_4_	1	1	0.5	0.5	1
*D*_5_	1	0	0	0	0.5

**Table 5:** Costs and various labor comparisons of light-duty cableway and road-skidding methods/(yuan).

Skidding methods	Cutting area code	Working quantity ready	Ready cost	Skidding quantity for ready	Skidding cost	Total working quantity	Fuel cost	Equipment depreciation cost	Day efficiency (m^3^/man)	Year efficiency (m^3^/man)	Unit cost	Cableway skidding savings rate
Road-skidding	122-1-3	158.00	16.26	1161.20	119.47	1319.2	7.71	5.00	0.74	185.74	148.44	29.50%
Cableway-skidding	122-1-3	120.00	12.35	747.10	76.86	867.1	9.96	5.48	1.12	281.12	104.65	

In addition to the total working quantity, the data are calculated by per m^3^ volume. “Working quantity ready” includes skidding road and setting up camps and cableways stages.
